# Draft Genome Sequence of *Chlamydia trachomatis* Strain 54, Isolated from the Urogenital Tract of a Male in Japan

**DOI:** 10.1128/genomeA.01242-15

**Published:** 2015-10-22

**Authors:** Tomohiro Yamazaki, Junji Matsuo, Momoka Kikuchi, Kentaro Miyamoto, Kentaro Oka, Motomichi Takahashi, Satoshi Takahashi, Torahiko Okubo, Hiroyuki Yamaguchi

**Affiliations:** a Department of Medical Laboratory Science, Faculty of Health Sciences, Hokkaido University, Sapporo, Japan; b Research Fellow of Japan Society for the Promotion of Science, Tokyo, Japan; c Miyarisan Pharmaceutical Co., Ltd., Tokyo, Japan; d Department of Infection Control and Laboratory Medicine, Sapporo Medical University School of Medicine, Sapporo, Japan

## Abstract

We report the draft genome sequence of *Chlamydia trachomatis* strain 54, isolated from the urogenital tract of a male in Japan, with unique polymorphic membrane proteins. Detailed genomic analysis will aid our understanding of the selective pressures that lead to sexual differentiation in chlamydial adaptive evolution.

## GENOME ANNOUNCEMENT

Obligate intracellular bacterium *Chlamydia trachomatis*, the causative agent of a sexually transmitted disease, can lead to serious medical complications such as infertility through ductal obstruction or pelvic inflammatory disease in females and testicular atrophy or epididymitis in males (1–6). Although chlamydial infection is reported in both men and women, the male urogenital tract lacks the microbial flora present in the female genital tract. This fact suggests that *C. trachomatis* strains would face different selective pressures, leading to specific genetic polymorphisms depending on sexual differentiation. However, *C. trachomatis* research has been limited to strains isolated from males because of difficulties in the isolation of organisms.

It is well established that the polymorphic membrane protein (Pmp) family generally possesses the properties of type V secretion systems (7). Interestingly, accumulating evidence has shown that among the nine Pmp families (A through I) expressed by *C. trachomatis*, PmpF is a high-definition phylogenetic marker that is strongly affected by host-selective pressures, such as those exerted by the microbial flora (7–9). In fact, we have recently demonstrated that PmpF of *C. trachomatis* isolated from the urogenital tracts of Japanese males showed less genetic diversity than the same protein isolated from the genital tracts of Japanese females, raising the idea that genetic diversity among *C. trachomatis* strains may be dependent on sexual differentiation (10). We therefore sequenced *C. trachomatis* strain 54 (*ompA* genotype B, accession number LC031846) isolated from the urogenital tract of a Japanese male.

The draft genome of *C. trachomatis* was obtained using an Illumina Miseq sequencer (Illumina, San Diego, CA, USA), with sequencing runs for paired-end sequences. The bacterial DNA libraries were prepared using an NEBNext DNA Library Prep master mix set for Illumina (New England Biolabs, Ipswich, Massachusetts, USA). The genome was assembled using *de novo* sequence assembler software (Platanus 1.2.1) (11), and 14 contigs were obtained with sizes ranging from 226 to 253,058 bp. Rapid Annotation using Subsystem Technology (RAST: http://rast.nmpdr.org/) was used for gene annotation (12). Functional annotation was performed using the Kyoto Encyclopedia of Genes and Genomes (KEGG) (http://www.genome.jp/kegg/) (13).

The draft genome sequence of *C. trachomatis* strain 54 was 1,046,714 bp in length (G+C content, 46.37%; coverage, 300-fold). The genome sequence contained 976 coding sequences with 38 tRNAs and 2 ribosomal RNAs. Using KEGG analysis and comparisons with *C. trachomatis* strain D/UW-3/CX (female strain) (NC_000117.1), the male strain was confirmed to possess conserved central carbon metabolic pathways and type III general secretion gene clusters. BLAST pairwise alignments interestingly revealed that the *pmpB* sequence of *C. trachomatis* strain 54 was unique, as well as the *pmpF*, a characteristic not previously reported for pathogenic *Chlamydiae*. A more detailed analysis of the genome will help us to understand the selective pressures that lead to sexual differentiation in chlamydial adaptive evolution.

### Nucleotide sequence accession numbers.

The draft genome sequence of *C. trachomatis* strain 54 has been deposited in the DDBJ database under accession numbers BCAM01000001 through BCAM01000014 (14 entries). The version described in this paper is the first version.
